# Primary Care Implementation of Genomic Population Health Screening Using a Large Gene Sequencing Panel

**DOI:** 10.3389/fgene.2022.867334

**Published:** 2022-04-25

**Authors:** Robert S. Wildin, Christine A. Giummo, Aaron W. Reiter, Thomas C. Peterson, Debra G. B. Leonard

**Affiliations:** ^1^ Department of Pathology & Laboratory Medicine, University of Vermont Health Network and Robert Larner M.D. College of Medicine at the University of Vermont, Burlington, VT, United States; ^2^ Department of Pediatrics, University of Vermont Health Network and Robert Larner M.D. College of Medicine at the University of Vermont, Burlington, VT, United States; ^3^ Department of Family Medicine, University of Vermont Health Network and Robert Larner M.D. College of Medicine at the University of Vermont, Burlington, VT, United States

**Keywords:** genomic medicine, population health, primary care, pilot implementation, screening, implementation research framework, real-world, clinical pilot

## Abstract

To realize the promise of genomic medicine, harness the power of genomic technologies, and capitalize on the extraordinary pace of research linking genomic variation to disease risks, healthcare systems must embrace and integrate genomics into routine healthcare. We have implemented an innovative pilot program for genomic population health screening for any-health-status adults within the largest health system in Vermont, United States. This program draws on key research and technological advances to safely extract clinical value for genomics in routine health care. The program offers no-cost, non-research DNA sequencing to patients by their primary care providers as a preventive health tool. We partnered with a commercial clinical testing company for two next generation sequencing gene panels comprising 431 genes related to both high and low-penetrance common health risks and carrier status for recessive disorders. Only pathogenic or likely pathogenic variants are reported. Routine written clinical consultation is provided with a concise, clinical “action plan” that presents core messages for primary care provider and patient use and supports clinical management and health education beyond the testing laboratory’s reports. Access to genetic counseling is free in most cases. Predefined care pathways and access to genetics experts facilitates the appropriate use of results. This pilot tests the feasibility of routine, ethical, and scalable use of population genomic screening in healthcare despite generally imperfect genomic competency among both the public and health care providers. This article describes the program design, implementation process, guiding philosophies, and insights from 2 years of experience offering testing and returning results in primary care settings. To aid others planning similar programs, we review our barriers, solutions, and perceived gaps in the context of an implementation research framework.

## 1 Introduction

We exist at the intersection of advances in genomics technology and quality, rapidly growing knowledge of the genetic underpinnings of human disease and susceptibilities, systems to support quality, and trending emphasis on maximizing preventive care opportunities. This frames an opportunity to realize a research-enlightened model of genomics-informed preventive healthcare.

Efforts to implement healthcare innovations often fail in the real world, even when research data supports their widespread use (Damschroder et al., 2009). Demonstrating feasibility of implementing genomic population health screening in a healthcare setting is a core challenge (Murray et al., 2018; Murray et al., 2021). Failures may occur for many reasons. Since many implementation barriers may be anticipated, frameworks for planning and evaluating implementations have been developed to facilitate informed planning and stimulate more implementation successes (Ginsburg et al., 2019; King et al., 2020). Implementation frameworks may be used during planning and executing implementations and when evaluating outcomes. Different frameworks have unique strengths (Roberts et al., 2019; King et al., 2020).

The Consolidated Framework for Implementation Research (CFIR) is a flexible option, whose creators derived five major domains from earlier healthcare implementation frameworks and theories: inner and outer settings, the individuals involved, the process, and the intervention (Damschroder et al., 2009). It defines within each domain distinct theoretical constructs that correspond to key success ingredients for each domain. CFIR’s inner and outer settings and the individuals involved domains constitute the implementation context. Constructs probing the motivations and rationale reside in the outer setting, while the characteristics of an organization, like culture, structure, readiness, and priority, comprise the constructs of the inner setting. CFIR refinements for implementing genomic medicine have been proposed (Orlando et al., 2018).

We report here the successful implementation of clinical genomic population health screening in primary care outpatient settings affiliated with a regional academic medical center in a rural US state. Key goals of the pilot intervention are listed in Table 1. To assist others considering similar efforts, our implementation is described here using a CFIR-based implementation science framework.

**TABLE 1:** Key goals of the genomic population health pilot implementation program.

Demonstrate the Feasibility of a Real-world genomic population health program with primary care at the center and genomics expertise in the background
Provide adult primary care patients of any health status and their providers with information about and access to a novel healthcare intervention built on prior genomics and genomic medicine research
Formulate and put into practice an accessible, one-page clinical informed consent form for genomic population health screening
Mimic conditions of recommended population health screening programs including no cost to patients for testing
Reduce or eliminate cost barriers for related genetic counseling (in-person or telemedicine), family member “cascade” testing for the health risks, and for reproductive partners of those with identified recessive carrier status
Incorporate scalability and existing workflows into the design, where possible, and identify opportunities and strategies for future improvements
Primary testing occurs in a Clinical Laboratory Improvement Amendments (CLIA) regulated laboratory using validated gene sequencing and confirmation methods
Define recommended responses to positive results in advance in the form of evidence-based Care Pathways designed by clinical specialists, communicated by written action plans, and activated by primary care providers
Provide patients and their providers with likely pathogenic and pathogenic germline variants in the context of information and suggested actions to address health and reproductive risks, using appropriate language
Clinical genomic population health test reports are treated like any other health information, placed in the patient’s secure electronic health record, and provided to patients
Patients and their primary care providers can work together to incorporate personal, social, and other health context into a responsive care plan
Provide updated reports and clinical updates whenever variant pathogenicity is reclassified

## 2 Context

The context of an implementation has great bearing on its likelihood of success. This report describes our implementation using CFIR domains. We are guided by each domain’s CFIR constructs (Damschroder et al., 2009; Orlando et al., 2018; King et al., 2020) without explicitly decomposing to them.

### 2.1 CFIR Outer Setting

The screening pilot occurs in Vermont, United States. Vermont is among the few states making strides toward healthcare reform with emphasis on value-based care (Grembowski and Marcus-Smith, 2018; Kissam et al., 2019). The focus signals openness to investment in innovative health prevention activities. Vermont’s accountable care organization (ACO), OneCare Vermont, is facilitating the transition to value-based care models. Federal, state, and private health insurers contract with the ACO and enrolled providers for a risk-adjusted, quality-focused, single annual payment for healthcare services. Alignment with the ACO allows better visibility into the real-world health impacts of innovations in population health screening.

Research involving return of actionable genomic sequencing results to patients for clinical use (Duow and Marjanovic, 2016; Linderman et al., 2016; Sanderson et al., 2016; Suckiel et al., 2016; Ryan et al., 2017; Murray et al., 2018; Rego et al., 2018; Reuter et al., 2018; Sapp et al., 2018; Schwartz et al., 2018; David et al., 2019; Nussbaum et al., 2019; Williams, 2019; Zoltick et al., 2019; Walton et al., 2020; David et al., 2021; Kelly et al., 2021; Khoury and Dotson, 2021; Lemke et al., 2021; Miller et al., 2021), potential harms of proactive testing, quality of next generation sequencing technology, and implementation of genomic medicine (Weitzel et al., 2016; Ginsburg et al., 2019; Williams, 2019) strongly informed our design.

Primary care is not a traditional setting for genetic testing or screening. Primary care providers do order pre-conception and prenatal screens and sample for newborn screening. Genomic literacy and competency among primary care providers is limited outside those areas. Upon receiving a positive genetic screening result, primary care providers’ responsive actions may be limited to patient notification and referral to a relevant specialist, or to following scripts, such as those provided by newborn screening laboratories. In general, time is the most limited resource for primary care providers and their staff. At the same time, risk assessment and directing and managing preventive care, the main objectives of genomic population health, occurs principally in the primary care setting.

Professional guidelines and resources for actionability of results, including the ACMG secondary findings guidance (American College of Medical Genetics and Genomics, 2019; Directors of the American College of Medical Genetics and Genomics, 2019; Nussbaum et al., 2019), ClinGen expert assessments (Rivera-Muñoz et al., 2018), and locally sourced specialty specific guidance, served as anchors for the design. However, updated non-genetics specialty practice guidelines are scarce for many of the health risk genes or are based on data from patients screened because of affected family members, often after an affected member had a positive indication-based test result. Current breast cancer genetic testing guidelines fail to identify almost half of individuals with a breast cancer risk gene pathogenic or likely pathogenic variant (Beitsch et al., 2019).

Clinical genetics laboratories now have extensive experience classifying the pathogenicity of gene sequence variations according to standardized systems (Richards et al., 2015; Nykamp et al., 2017) and linking variants to peer-reviewed literature supporting clinical validity. New variant and clinical validity/utility information evolves and justifies re-classification of variants with a necessity to update clinical reports. Nonetheless, evidence is lacking to accurately classify much of the human genomic sequence variation as pathogenic, benign, or likely so. For these variants of unknown/uncertain clinical significance (VUSs), it is not currently known whether they impact health.

Anecdotal reports describe missed, inappropriate, and or unnecessary medical responses after genetic or health-risk testing. These have been used to warn against broad-based genomic screening at population scale (Murray et al., 2018). Restricted genetic competency among non-geneticists tasked with interpreting genetic test results may facilitate insufficient responses even when preventive opportunities exist. Genetic disease expertise clearly has a role in population genomic screening (Lemke et al., 2021).

The popularity of consumer-oriented genomic testing and concerted efforts to increase the genomic literacy of Americans has fostered growing public awareness of links between heritable genetic variation and disease. Programs that performed health-related genomic screening tests for physicians and health administrators have helped them personally identify with the potential for routine genomic risk screening and raised awareness and interest among non-genetic specialists and primary care leaders (Briggs, 2016; Masterson, 2016).

At the same time, widespread testing has raised concern regarding the privacy of genetic information, genetic discrimination, as well as the commoditization of genetic data. Many people are unaware that a genetic result obtained outside of a healthcare setting is not subject to HIPAA privacy law protections nor CLIA laboratory quality certification, and many lack clarity about the extent of protections against genetic discrimination provided by federal and state laws.

Information relevant to a patient’s health is recorded in the health record. Yet electronic health records (EHRs) generally lack robust, expansible, accessible, and readily implementable functions to store, annotate, retrieve, and update germline genetic information and annotations that may remain clinically relevant for many decades (Walton et al., 2020).

While large cohorts of research participants have received exome or genome sequencing results, fully clinical programs screening large numbers of health risk genes have until recently been offered only in clinics catering to self-pay clients. Research screening programs are being adapted to a clinical model.

### 2.2 CFIR Inner Setting

The University of Vermont Medical Center (UVMMC) is a regional academic tertiary care center serving a largely rural population in Vermont and northern New York, where Northern-European ancestry and white race are claimed by most of the population. UVMMC is the academic teaching hospital of the UVM Health Network that includes five other rural hospitals, home health and hospice, a physician organization, and collaboration with a Federally Qualified Health Center. By the end of 2022, all will operate on UVMMC’s Epic Systems EHR instance. UVMMC and Network partner Porter Hospital have multiple community primary care clinics in Chittenden and Addison counties, VT.

Traditional models of genetic disease detection and prevention are practiced, including mandated newborn screening, variable documentation of family health history, genetic specialist evaluation, genetic counseling, and genetic testing of individuals and families at risk or manifesting genetic conditions. No DNA-based primary screening of people without risk factors occurs. Individuals at higher risk of genetic predisposition due to a diagnosis of colon or endometrial cancer are screened for Lynch Syndrome using immunohistochemistry. Individuals with a family history suggesting predisposition to cancer may be referred to the Familial Cancer Program of genetic oncologists and genetic counselors.

An on-site Genomic Medicine Laboratory, directed by molecular pathologists, two Ph.D. molecular biologists, and a clinical and laboratory geneticist, performs NGS sequencing of tumor DNA and RNA for precision oncology therapy. All clinical germline testing is sent to referral laboratories.

UVMMC has a robust Patient and Family Advisors (PFA) program (Celenza et al., 2017; Wahlberg et al., 2021). PFAs are volunteers invited to provide patient- and family-centered perspectives to implementation teams during project planning.

UVMMC health information technology (HIT) resources are extensive yet principally focused on business operations and dissemination of Epic Systems products throughout the health system.

### 2.3 CFIR Characteristics of Individuals and Implementation Roles

The pilot was envisioned, designed and supported by the Chair of the Department of Pathology and Laboratory Medicine (DGBL), a molecular pathologist who founded the Genomic Medicine Program. The Genomic Population Screening Program implementation was led by an ABMG Clinical Geneticist (RSW) with both laboratory and patient care expertise. Both (DGBL and RSW) have been involved in national efforts to promote realization of the genomic medicine potential in health care. The geneticist has broad experience in genetic and genomic medicine, including solo genetics practice in rural and suburban areas, academic and non-academic settings, workforce training, education, policy, clinical molecular genetics, and rare disease research. The Chair of Family Medicine (TCP) and the Family Medicine champion provider (AWR) both had professional experiences arising from the Illumina “Understanding Your Genome” (UYG) program performed locally in 2017 that informed their participation and commitment (Briggs, 2016; Masterson, 2016). Both Chairs are leaders at the UVMMC and the UVM Health Network with access to health system leaders.

An experienced clinical and laboratory certified genetic counselor (CAG) helped plan and execute the pilot, she provided the genetic counselor’s perspective and performed genetic counseling. A second genetic counselor provided temporary, part-time support.

Three retired non-medical professionals from the community who volunteer as PFAs provide input during both planning and execution phases. Ten PFA volunteers contributed as a group to develop a new written clinical consent form, as well as a brief animated video providing a patient-oriented overview of the program.

Participating PCPs largely belong to two multi-site family medicine practice groups of the UVM Health Network Medical Group. Most were recruited informally by the PCP champion and other participating PCPs, while a few were approached by knowledgeable patients. Most are physicians, but nurse practitioners and physician assistants also participate. None received participation incentives.

Patients offered the test must meet these eligibility criteria: at least 18 years old, they and their partner are not pregnant, their PCP participates in the pilot and received program training, and the patient is attributed to Vermont’s ACO. There are no restrictions based on health status, family history, or other health risk factors.

### 2.4 CFIR Implementation Process

#### 2.4.1 Planning for Implementation

An approximately 1 year long planning process occurred prior to offering the first test. Test information, engagement materials, and a mandatory consent form were developed, implementation partners were engaged and contracted, and care pathways were designed for those conditions having the highest expected follow-up need after a positive test. For most providers and staff contributing to the planning and early implementation phases, a portion of usual salary was paid.

Planning culminated in a business plan approved by UVMMC leaders. It communicated the project’s scope, model, justifications, and expected or potential impacts on the institution’s operations and employees as well as patients. No UVMMC funding was requested.

#### 2.4.2 Legal, Compliance, and Ethics

Legal and compliance considerations arise from making this an extension of clinical care. Using a CLIA-certified laboratory and working within HIPAA and other health statutes and regulations is essential. The protections and limitations of the Genetic Information Nondiscrimination Act and Vermont’s additional non-discrimination statutes are emphasized when educating patients about potential testing risks. An M.D. medical ethicist and the health system’s legal counsel guided our decisions regarding ethically and legally important issues. ACOs are permitted to issue waivers for innovative care programs. We obtained such a waiver to permit us to legally offer the screening test and associated genetic counseling at no cost.

### 2.5 CFIR Intervention Characteristics

#### 2.5.1 Guiding Principles

We developed a set of principles that guided us as we designed and modified the program. These included implementation as a clinical pilot program, taking pains to avoid mischaracterization as a research study. The need for simplicity and practicality in a contemporary clinical environment was essential. Like other preventive health testing, patient participation is voluntary and with clinical informed consent and results are placed in the EHR. Health condition and risk information need to be available to providers. Genetic test reports convert genetic testing results into health preserving actions. Patients and providers have varying capacities and tolerances for information complexity and benefit from ready access to experts.

We placed primary care at the center of the patient activity because that is where most preventive health screening and day-to-day health risk management occurs. In addition, patients generally have a trusting relationship with their primary care provider. Specialists are included in the program for referral of patients with actionable results best managed by the most expert healthcare available. Because we envision the program as a pilot for widespread genomic population health screening, we strive for scalability in the program’s elements, communications, and workflows.

We support testing and general genetic counseling at no cost so that lack of financial resources does not prohibit access. We wish to demonstrate the feasibility and character of population-based screening that will eventually be included in value-based insurance benefits, and include Vermonters who are not necessarily healthy, wealthy, or employed. Lack of need for billing also simplified implementation.

#### 2.5.2 Genetics Practice, Laboratory Experience, and Administrative Location

The participation of a clinical geneticist and genetic counselor, both with molecular laboratory experience, and a molecular pathologist and laboratory founder, provided perspective on how the design interfaced with traditional medicine, medical genetics, and external partners. Locating the program administration in the clinical Genomic Medicine Laboratory leveraged the broad multi-specialty and primary-care connections of the hospital laboratory as well as infrastructure for contracting with reference laboratories.

#### 2.5.3 Care Pathways

To address concerns about inappropriate use and shortage of definitive guidelines for genes in our panel, we worked with physician specialists in cardiology and hereditary cancer to design evidence-informed care pathways. A Care Pathway Work Group chaired by the geneticist was established for this purpose. As the testing workflows and care provided after positive test results impacts patients, PCPs, and staff, each contributed representatives to the Care Pathway Work Group in addition to specialty members. Three PFAs joined this group and were instrumental in the development of pre-test Care Pathways for introducing the test to patients and to inform and educate them prior to deciding whether to test, as well as discussing the post-test results disclosure pathways.

Other members of the work group include the PCP champion, a nurse-administrator champion, and the genetic counselor. During the planning phase, a family medicine practice director, a cardiologist specializing in electrophysiology, a genetic oncologist, and an M.D. medical ethicist participated.

Specialty Care Pathways describe specific steps for responding to positive test results in certain genes, including which clinical correlation tests the PCP may order, the specialty referral criteria, and anticipated tests that may be done for staging and screening during evaluation by a specialist. Evidence-informed Care Pathways for genes in other specialty areas are designed by the clinical geneticist in consultation with published literature and local specialists as relevant test results occur.

#### 2.5.4 Use of Existing Systems

Implementation is easier when existing systems can be incorporated in the design. We leveraged primary care’s models of annual wellness visits and continuity of care to place novel testing in an existing practice framework. A well-established laboratory send-out workflow facilitated partnering with a commercial laboratory instead of onsite testing and germline variant interpretation. Patients with results suggesting cancer predisposition are referred to the existing Familial Cancer Program. Our model for providing free, test-related general genetic counseling evolved. The pragmatic solution was contracting with our Clinical Genetics service for patient- and provider-driven genetic counseling requests. We did not leverage any potential EHR functionality that required customization or a “build,” as we lacked access to the necessary HIT resources during the reported-on period.

#### 2.5.5 Avoiding Confusion With Traditional Genetic Screening and Evaluation Paradigms

We characterize this program as genomic population health screening. Pains are taken to emphasize that this new test should not replace existing indication-based genetic evaluation and testing. Nor should it replace existing genetic disease or risks screenings, like newborn screening and pre-conception/pre-natal carrier testing. Patients with personal or family history indications for medical genetic evaluation are asked to utilize existing specialty care services for those needs. However, because this screening test has the potential to identify *unrelated* hidden health risks, patients are not excluded for having a genetic testing indication, an existing genetic diagnosis, nor any other diagnosis.

An important distinction from indication-based genetic testing is that variants of unknown significance (VUSs) are not reported in the program’s screening test. This is because the prior probability of many screened-for conditions is assumed to be zero in the tested population because they are not selected for any phenotype (Murray et al., 2018). This is an important educational topic for PCPs.

## 3 Detail

### 3.1 The Testing Process (The Intervention)

#### 3.1.1 Test Information and Offering the Test

During pre-visit planning meetings, PCPs, and staff identify eligible patients. Testing is offered to those by their primary care provider during usual care. This may occur at an annual wellness visit, or at any other visit where discussing the test does not interfere with the visit’s primary focus. Non-physician staff may inform patients that a new screening test is available. They may play the 1-min and 46-second-long animated overview video. PCPs develop brief scripts which they feel help introduce the test to patients.

**FIGURE 1:**
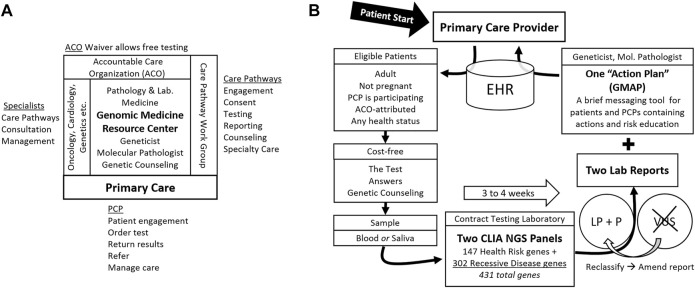
Genomic Population Health Pilot Program: Organization and Testing Process. **(A)** The multi-disciplinary team, its interfaces, and team member activities. The Genomic Medicine Resource Center provides support for primary care providers (PCP) and patients and coordinates the team. **(B)** The testing process and test details. Patients engage first with their primary care provider (PCP). Arrows indicate steps in the process. Abbreviations: Electronic health record (EHR), accountable care organization (ACO), Clinical Laboratory Improvement Amendments (CLIA), next generation sequencing (NGS), likely pathogenic (LP), pathogenic (P), variant of uncertain significance (VUS), and Genomic Medicine Action Plan (GMAP).

A folder given to the patient contains written information about the test at multiple levels of depth as well as key forms. This “patient packet” contains a tri-fold brochure, a 6-page “Frequently asked Questions (FAQ)” document, a list of genes covered by the test, the hospital-approved one-page clinical consent form, and “next-steps” instructions describing sample collection options and logistics. Each of these contains contact information for the Genomic Medicine Resource Center (GMRC) (Figure 1A), where questions are answered by a geneticist or genetic counselor for free, and where formal pre-test genetic counseling is arranged on request. For the PCP’s convenience, the required send-out test order forms, customized for the test, are also included in the patient packet. The public web page offers the video and downloadable patient packet materials (Supplementary Materials).

Patients review the information and ask questions of the PCP. PCPs refer patients with genetic or logistical questions, or those taking more time, to the GMRC. Patients may decide to proceed with the test immediately or take time to review and decide. Those deciding to test must sign the consent form which is scanned into the EHR before an order can be entered. A blood or saliva sample is obtained and shipped to the testing laboratory by the UVMMC clinical laboratory along with the testing laboratory’s requisition completed by the PCP.

#### 3.1.2 Performing the Test

The testing laboratory accessions requisitions and samples. Orders are tracked locally by the GMRC staff using the testing laboratory’s secure online portal account dedicated to the program. We portray to patients a single test that may detect potential health risks for themselves and their family members. At the testing laboratory, this consists of two standard NGS gene panels. The first 147 gene panel is a “Pro-active” health screen for monogenic cancer and cardiovascular risks as well as some relatively common recessive risks (Haverfield et al., 2021). The second, 302-gene panel is a “Comprehensive Carrier” screen for monogenic recessive disorders. The panels overlap, so the union of genes sequenced is 431 (Wildin, 2019). Turnaround time is three to 4 weeks.

#### 3.1.3 Preparing and Augmenting the Results for Action

The testing laboratory’s results are reported in multipage PDF documents, one for each gene panel. GMRC staff download reports from the secure portal. The reports contain information about the variants found, the diseases they are linked to, inheritance patterns, and, in some cases, notations regarding reduced penetrance. The basis for variant classification as Likely Pathogenic (LP) or Pathogenic (P) using the testing laboratory’s variant classification system (Nykamp et al., 2017) is included. Variants of uncertain significance (VUSs) are not reported. If VUSs are subsequently reclassified as LP or P, the testing laboratory issues an amended report with the new or classification-altered variants.

The GMRC staff reviews the testing laboratory’s reports and produces a templated “Genomic Medicine Action Plan” (GMAP) messaging document (manuscript in preparation). Briefly, the one-to three-page GMAP is designed to focus provider and patient attention on the actionability of the results. Another function is to limit inappropriate responses to the results. The GMAP suggests PCP and patient actions and education and notes appropriate care pathways. The GMAP is pre-pended to the two report PDFs and the three documents merged. This augmented report is placed in the EHR as the original test order is finalized and PCPs are notified.

#### 3.1.4 Returning Results to Patients and Genetic Counseling

PCPs receive guidance from the GMRC on how to return results and discuss them with patients; however, they develop their own protocols for how this is done in their practice. PCPs may perform clinical correlations to refine the risk for any positive results guided by the GMAP, such as reviewing personal and family health histories and ordering additional testing, procedures, and or referrals.

Post-test general genetic counseling is offered at no cost and is encouraged to discuss any results, especially in complex scenarios. Patients referred to the Familial Cancer Program receive genetic counseling during that billed specialty visit. For referral to other specialties lacking their own genetic counselors, a no-cost genetic counseling visit is strongly encouraged before the specialty visit. Genetic counseling is available in person or via tele video.

#### 3.1.5 Family Member and Partner Testing

The information resulting from individual screening is useful to family members and to couples who may become pregnant. The GMAP messaging urges patients to review the full test reports that contain information about recessive disease risk, inheritance patterns, family member testing, and partner testing. It encourages patients to share the results with family members and briefly summarizes inheritance risks. The testing laboratory offers no-cost testing of blood relatives within 90 days of the report for any positive result on the “Pro-active” panel. The GMAP also suggests reproductive partner testing where appropriate and highlights low-cost partner testing offered by the testing laboratory. Genetic counseling is recommended in conjunction with both family member and partner testing. However, this pilot program does not manage family member or partner testing.

### 3.2 Summary of Testing Experience

Testing began 1 November 2019, in one Family Medicine practice with one PCP champion. Additional PCPs and practices joined as roll-out issues were resolved and as clinic workloads permitted. By March 2020, four additional PCPs and one additional clinic site were offering testing. Nearly all patients were tested by a Family Medicine PCP. The remainder were Internal Medicine patients who heard about and requested the test. Since patients are offered testing in the context of primary care visits, they reflect the demographics and health status of individuals frequenting primary care offices.

Two years after testing began, twenty different PCPs had ordered at least one test. One quarter of the providers ordered three quarters of the tests. 186 patients between 18 and 92 years old had been tested. Median age was 58. Thirteen percent of tests had no reportable variants. The rest reported one or more dominant or recessive likely pathogenic or pathogenic variants.

### 3.3 Adapting to Changes in Outer Setting

**FIGURE 2:**
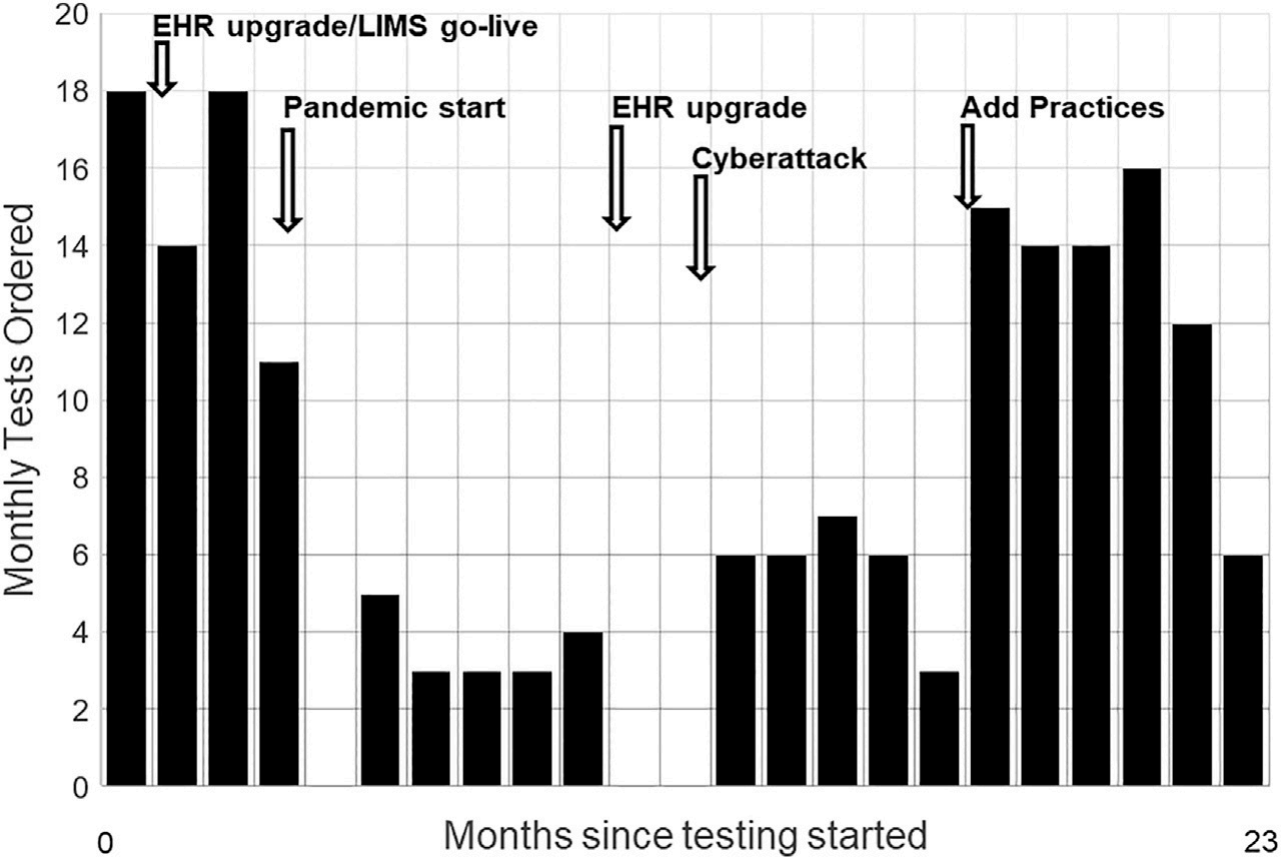
Monthly Test Volumes and Key Events. Monthly test volumes during the first 23 months of testing, starting November 2019. Disruptive events included major upgrades to the electronic health record (EHR), replacement of the hospital’s laboratory information system (LIMS), the onset of the COVID-19 pandemic, an EHR upgrade requiring widespread staff training, and a cyberattack that took all information systems offline for weeks. Adding a second practice group with its own physician champion increased volumes.

Figure 2 shows monthly tests and the sources and timing of unanticipated inner setting demands on primary care and Genomic Medicine. Operational disruptions from the COVID-19 pandemic interrupted testing for about 2 months. Staffing issues quieted hoped-for expansion of the perceived optional activity to more primary care providers. We built a public web page where patients engaged through telemedicine visits can view the animated educational video, and download test information, educational resource documents, and the consent form, including contact information for the GMRC (Wildin, 2020). A home saliva sampling kit option was also added.

In response to laboratory wide needs, an HIT systems architect was engaged. This experienced professional performs a critical adaptor function to the HIT operations and prevailing culture of our setting.

### 3.4 Quality Assessment

To assess early patients’ perceptions of the program’s implementation effectiveness and to focus quality improvement efforts, in June 2020 we mailed a two-page survey to the first 61 patients tested along with a postage-paid return envelope. After two reminder letters, 19 surveys were returned. One was blank and excluded from tabulation. The Supplementary Material shows 18 tabulated responses in the survey instrument format. Aside from logistical challenges like receiving printed results in the mail, which we worked to improve, the survey indicated general satisfaction or enthusiasm about the testing design and process, and for the value proposition. Of note, patients strongly endorsed that the PCP’s office is the right place to offer this testing.

Table 2 describes events captured by our quality surveillance processes and how we responded. Most resemble those occasionally encountered in health care and none affected patient health. While data about the rate of patients choosing to test when offered testing, and why patients declined testing, are potentially informative, their collection was not practical during this pilot implementation.

**TABLE 2:** Notable Events. Ongoing quality surveillance identified refinement opportunities.

Event	Count	Response
The test was ordered in error. Quality surveillance identified lack of a signed consent. Testing was halted, the order was cancelled, and results were neither recorded nor released	1	PCPs were instructed not to “pend” orders while a patient considers whether to test
A patient complained because they received a bill for indicated professional services for an identified health risk	1	Although the limits of cost-free test-related services are delineated in the pre-test patient information, the importance of timely reminders during the patient journey is now emphasized
A patient with an anxiety disorder complained to their PCP of increased symptoms during testing and immediately after result delivery. The PCP successfully managed the transient exacerbation	1	Onboarding education cautions about timing of testing for patients with active mental health concerns are further emphasized
Report made to the health system’s risk reporting system	None	None
Signature or manual data entry errors involving paper test requisitions or paper consent forms	∼5%	Communications to correct each. Provider and staff re-education, and continued pressure for EHR integration resources

## 4 Discussion

### 4.1 Conclusion

The key goals of this pilot implementation of clinical genomic population health screening of any-health-status adults were accomplished (Table 1). This demonstrates the feasibility of translating lessons from prior population sequencing and return of results research into clinical practice, which was the primary goal. Key differentiators of our implementation include placing primary care at the center, using a large, pre-defined clinically relevant target gene panel performed in a clinical laboratory, offering testing as part of usual preventive care at no cost, providing a written action plan with the test reports, and not being a research protocol.

The implementation we describe here leveraged all the opportunities and overcame most of the challenges cited for “non-traditional genetic testing” in the American College of Medical Genetics and Genomics’ “Points to Consider” analyses, including the important roles primary care providers contribute (Bean et al., 2021; Murray et al., 2021). Strengths included leadership engagement with tools like a personal genomics test that occurred years prior to beginning the pilot, getting formal buy in from medical center administration with a non-financial business plan, involving diverse stakeholders in the design and implementation process and making it worth their time, and leveraging existing workflows wherever possible.

We contracted for existing validated tests and primary reporting with a commercial laboratory. This allowed us to move forward sooner and with less expense than if we had to implement germline testing, variant interpretation, and reporting ourselves.

Indirect measures of success include that new PCPs continue volunteering, most PCPs involved have continued to offer the test, and patients continue to get tested. Notably, no participation incentives are provided to the PCPs. Recruiting new PCPs was actively limited due to unrelated staff shortages and suspended during the COVID-19 public health crisis, redemonstrating the susceptibility of new prevention-oriented programs to externally imposed prioritization.

Patient complaints are few, are related to process and communication, and are easily addressed. Unanticipated resource demands have not surfaced, and no critical element of the complex multi-disciplinary design has failed or had to be withdrawn. Our patient quality survey is a direct measure addressing some of the same data types as the survey by Orlando et al. (2018). The results are generally positive and support the assertion that the process is sufficiently patient-centric.

Barriers to scaling up are common in new interventions. We underestimated the need for leadership engagement in HIT and the relative priority for planned system-wide HIT transformation, where tension for change was far higher. HIT resources were unavailable to build consent, order, and resulting experiences familiar to the PCPs. The EHR-plus-paper order process we used instead burdens clinic staff and dissuaded some PCPs from participating. This adaptation is also the principal source of tracked process errors. EHR-based improvements will be prioritized once the system-wide Epic implementation is completed in April 2022. A separate, secure data system was built internally to track the multiple process steps. The solution allows oversight but is neither interfaced nor scalable. The criterion that tested patients are attributable in Vermont’s ACO was similarly challenging because ACO status is not reliably reflected in our EHR. It requires a manual inter-institutional lookup process.

The strong knowledge and experience of the principal implementers and of the primary care and other key partners, and the continued involvement of the PFAs, all contributed to resilience in the face of disruptive shifts in the setting that eluded anticipation, such as the COVID-19 pandemic and a UVMMC cyberattack.

### 4.2 Generalizability

The implementation of genomic population health screening in primary care at our institution benefited from elements in the outer setting, like the ACO, and in the inner setting, like engaged leaders who embrace innovation, champion providers, a highly collaborative team with broad expertise and capabilities, and availability of non-research funding of the pilot. While not unique, these advantages are not universal. Our guiding design principles may not be shared in every instance, and situations calling for pragmatism may also diverge. Presenting our implementation openly and in a recognized framework may help others identify their unique paths to success.

### 4.3 Future Directions

While not a goal of the pilot, we recognize that the patients’ clinical results combined with their personal and family health histories represent data types underlying a key phase of learning healthcare systems (LHS) (Schwartz et al., 2018; Williams et al., 2018). Having met our goal of demonstrating feasibility, we anticipate building a real-world LHS with related implementation, outcomes, return on investment, personal, educational, and health system research that can be combined or compared with similar data from other genomic population health screening programs.

We wish to increase testing for younger and healthy adults, who visit their PCP less often, by engaging them through EHR patient portal messages (Christensen et al., 2021) and by expanding testing to women’s health clinics. To accomplish enhanced risk assessment for genetic disease risks, family health history and genomic population health risk information should be co-analyzed (Wildin et al., 2021). This adds complexity but could propel family member (“cascade”) testing, an important added value for genomic population health screening.

Finally, since our pilot’s funding is finite, there is a need for both stable and scalable investment in this and similar programs that support the enhanced prevention focus of value-based care. We envision genomic population screening as a future benefit in value-based care payment contracts, supporting the preservation of a healthy state in both individuals and populations.

## Data Availability

The original contributions presented in the study are included in the article/Supplementary Material, further inquiries can be directed to the corresponding author.
