# Supplementary material for: Primary Care Implementation of Genomic Population Health Screening Using a Large Gene Sequencing Panel
Source: Front Genet. 2022 Apr 25;13:867334. doi: 10.3389/fgene.2022.867334 (PMC9081681; doi:10.3389/fgene.2022.867334)
Supplement: Supplementary file 1 [file DataSheet1.DOCX]

Supplementary Material

# Supplementary Table

Program quality survey results

Survey methods: In June of 2020, we mailed a quality improvement survey to the first 61 patients tested by the program. After two reminder letters, 19 surveys were returned. One was blank and was excluded from tabulation. The tabulated survey results are presented in the survey format below.

Instructions to recipients: “…we would appreciate your feedback to help us improve the program. If you would like to help, please answer our questions on the enclosed short survey and return it to us using one of the methods listed there. Voluntary completion of this 2-page survey will help us improve the Genomic DNA Test program. We may also publish the survey results in a medical journal without revealing individual answers.”

1. Thinking of the time you were offered the test, which information sources did you find helpful?

|  | not helpful | helpful | very helpful | did not view or do | *not answered* |
| --- | --- | --- | --- | --- | --- |
| Short animated video* | 0 | 2 | 1 | 12 | *3* |
| Brochure | 0 | 11 | 3 | 2 | *2* |
| FAQ document | 1 | 9 | 2 | 3 | *3* |
| Discussion with PCP | 0 | 9 | 9 | 0 | *0* |

1. Thinking of the time when you were deciding about testing, how much did these matter to you?

|  | not at all | somewhat | a lot | did not consider | *not answered* |
| --- | --- | --- | --- | --- | --- |
| Value for my own health | 0 | 6 | 11 | 1 | *0* |
| Value for my family’s health | 0 | 4 | 12 | 1 | *1* |
| Value for my provider | 1 | 8 | 7 | 2 | *0* |
| Value for researchers | 0 | 7 | 10 | 0 | *1* |

1. Thinking of the time when you were waiting for the test results, how did you feel?

|  | really short | about right | too long | Don’t remember |
| --- | --- | --- | --- | --- |
| The wait time seemed… | 1 | 13 | 0 | 4 |
|  | anxious | neutral | excited | Don’t remember |
| My emotions were… | 0 | 15 | 2 | 1 |

1. What happened when the test results were ready? **

|  | No | Yes | Don’t remember | *not answered* |
| --- | --- | --- | --- | --- |
| I got a call from my provider’s office | 3 | 14 | 1 | *0* |
| I received test result documents in the mail | 5 | 11 | 2 | *0* |
| I reviewed the test result documents | 2 | 14 | 0 | *2* |

1. Thinking of the time when you had received your results, …

|  | No | Yes | Don’t remember | *not answered* |
| --- | --- | --- | --- | --- |
| I was referred to a specialist because of my result | 13 | 3 | 1 | *1* |
| I contacted the Genomic Medicine Resource  Center for help understanding the results | 14 | 4 | 0 | *0* |
| I shared the results with family members | 7 | 9 | 0 | *2* |
|  | None | This many: | Don’t know | *not answered* |
| Family members got tested because of my results | 15 | 1 (3 tested) | 1 | *1* |

1. As of today, I feel that …

|  | Not really | Somewhat | Yes | *not answered* |
| --- | --- | --- | --- | --- |
| I need help understanding my results | 11 | 4 | 1 | *2* |
| I am satisfied with the testing and result process | 0 | 6 | 11 | *1* |
| The printed result summary and test report I received were clear and helpful | 3 | 6 | 7 | *2* |
| I would recommend others get this test | 1 | 6 | 10 | *1* |
| My primary care office is the right place to do this | 0 | 3 | 14 | *1* |

1. Please add comments that will help us improve the program. (7 responses, *paraphrased*)

- Satisfied with the process and have confidence in their provider
- Unexpected finding of a genetic disorder requiring treatment, crediting provider with handling it well and implementing an effective care path
- Two relevant results found. One they were already aware of because family member had died of the disorder, but the second one was a surprise.
- Happy that no cancer predisposition was found.
- Suggested mailing the test results and information to patients. *[Respondent answered “no” to section 4. “I received test results in the mail”].*
- Described the process as straightforward and very illuminating.
- Did not receive their report in the mail but at their next doctor visit.
- COVID-19 lockdown prevented return to provider for results. Requested genetic counseling. *[Genetic counseling was subsequently arranged].*

* The earliest patients were informed and tested before the short, animated overview video was available.

** Some patients received their results in the PCP’s office, so some did not receive a call about them, nor did they receive the printed copy *in the mail*. At least one did not receive the printed copy at all, and so could not review the printed results. This was subsequently provided.

***Supplementary Information***: link to patient-oriented and downloadable information about the test:

<https://www.uvmhealth.org/GenomicDNAtest>

***Gene Lists:*** Genes sequenced or otherwise assayed in the offered test are listed here:

**Health Risk Genes**

Cardiovascular Disease Risk ACTA2 ACTC1 ACTN2 ACVRL1 APOB BAG3 BMPR2 CACNA1C CACNB2 CALM1 CALM2 CALM3 CASQ2 CAV1 CAV3 COL3A1 CRYAB CSRP3 DES DMD DSC2 DSG2 DSP EMD ENG F2 F5 F9 FBN1 FHL1 FLNC GDF2 GLA GPD1L HCN4 JUP KCNE1 KCNE2 KCNH2 KCNJ2 KCNQ1 LAMP2 LDLR LDLRAP1 LMNA MYBPC3 MYH11 MYH7 MYL2 MYL3 MYLK NKX2-5 PCSK9 PKP2 PLN PRKAG2 PRKG1 PROC PROS1 RBM20 RYR2 SCN5A SERPINC1 SGCD SMAD3 SMAD4 TCAP TGFB2 TGFB3 TGFBR1 TGFBR2 TMEM43 TNNC1 TNNI3 TNNT2 TPM1 VCL

Cancer Risk APC ATM AXIN2 BAP1 BARD1 BMPR1A BRCA1 BRCA2 BRIP1 CDC73 CDH1 CDK4 CDKN2A CHEK2 DICER1 EPCAM FH FLCN GREM1 HOXB13 KIT MAX MEN1 MET MITF MLH1 MSH2 MSH3 MSH6 MUTYH NBN NF1 NF2 NTHL1 PALB2 PDGFRA PMS2 POLD1 POLE PRKAR1A PTCH1 PTEN RAD51C RAD51D RB1 RET SDHA SDHAF2 SDHB SDHC SDHD SMAD4 SMARCA4 SMARCB1 STK11 TMEM127 TP53 TSC1 TSC2 VHL WT1

Other Risks ATP7B CACNA1S HAMP HFE HJV OTC RYR1 SERPINA1 SLC40A1 TFR2

**Carrier Genes (a wide range of disorders)**

ABCB11 ABCC8 ABCD1 ACAD9 ACADM ACADVL ACAT1 ACOX1 ACSF3 ADA ADAMTS2 ADGRG1 AGA AGL AGPS AGXT AIRE ALDH3A2 ALDOB ALG6 ALMS1 ALPL AMT AQP2 ARG1 ARSA ARSB ASL ASNS ASPA ASS1 ATM ATP6V1B1 ATP7A ATP7B ATRX BBS1 BBS10 BBS12 BBS2 BCKDHA BCKDHB BCS1L BLM BSND BTD CAPN3 CBS CDH23 CEP290 CERKL CFTR CHM CHRNE CIITA CLN3 CLN5 CLN6 CLN8 CLRN1 CNGB3 COL27A1 COL4A3 COL4A4 COL4A5 COL7A1 CPS1 CPT1A CPT2 CRB1 CTNS CTSK CYBA CYBB CYP11B1 CYP11B2 CYP17A1 CYP19A1 CYP27A1 DBT DCLRE1C DHCR7 DHDDS DLD DMD DNAH5 DNAI1 DNAI2 DYSF EDA EIF2B5 ELP1 EMD ERCC6 ERCC8 ESCO2 ETFA ETFDH ETHE1 EVC EVC2 EYS F11 F2 F5 F9 FAH FAM161A FANCA FANCC FANCG FH FKRP FKTN FMR1 G6PC G6PD GAA GALC GALK1 GALT GAMT GBA GBE1 GCDH GFM1 GJB1 GJB2 GLA GLB1 GLDC GLE1 GNE GNPTAB GNPTG GNS GP1BA GP9 GRHPR HADHA HAX1 HBA1 HBA2 HBB HEXA HEXB HFE HGD HGSNAT HJV HLCS HMGCL HOGA1 HPS1 HPS3 HSD17B4 HSD3B2 HYAL1 HYLS1 IDS IDUA IL2RG IVD KCNJ11 LAMA2 LAMA3 LAMB3 LAMC2 LCA5 LDLR LDLRAP1 LHX3 LIFR LIPA LOXHD1 LPL LRPPRC MAN2B1 MCCC1 MCCC2 MCOLN1 MED17 MEFV MESP2 MFSD8 MKS1 MLC1 MMAA MMAB MMACHC MMADHC MPI MPL MPV17 MTHFR MTM1 MTRR MTTP MUT MYO7A NAGLU NAGS NBN NDRG1 NDUFAF5 NDUFS6 NEB NPC1 NPC2 NPHS1 NPHS2 NR2E3 NTRK1 OAT OPA3 OTC PAH PC PCCA PCCB PCDH15 PDHA1 PDHB PEX1 PEX10 PEX12 PEX2 PEX6 PEX7 PFKM PHGDH PKHD1 PMM2 POMGNT1 PPT1 PROP1 PRPS1 PSAP PTS PUS1 PYGM RAB23 RAG2 RAPSN RARS2 RDH12 RMRP RPE65 RPGRIP1L RS1 RTEL1 SACS SAMHD1 SEPSECS SERPINA1 SGCA SGCB SGCG SGSH SLC12A3 SLC12A6 SLC17A5 SLC22A5 SLC25A13 SLC25A15 SLC26A2 SLC26A4 SLC35A3 SLC37A4 SLC39A4 SLC4A11 SLC6A8 SLC7A7 SMARCAL1 SMN1 SMPD1 STAR SUMF1 TAT TCIRG1 TECPR2 TFR2 TGM1 TH TMEM216 TPP1 TRMU TSFM TTPA TYMP USH1C USH2A VPS13A VPS13B VPS45 VRK1 VSX2 WNT10A XPA XPC ZFYVE26

For more information on these genes and their related diseases, search the gene name above at https://ghr.nlm.nih.gov/ (Genetic Home Reference, a service of the National Library of Medicine)
